# Search for viral agents in cerebrospinal fluid in patients with multiple sclerosis using real-time PCR and metagenomics

**DOI:** 10.1371/journal.pone.0240601

**Published:** 2020-10-28

**Authors:** Karol Perlejewski, Iwona Bukowska-Ośko, Małgorzata Rydzanicz, Tomasz Dzieciątkowski, Beata Zakrzewska-Pniewska, Aleksandra Podlecka-Piętowska, Agata Filipiak, Krzysztof Barć, Kamila Caraballo Cortés, Agnieszka Pawełczyk, Marek Radkowski, Tomasz Laskus

**Affiliations:** 1 Department of Immunopathology of Infectious and Parasitic Diseases, Medical University of Warsaw, Warsaw, Poland; 2 Department of the Medical Genetics, Medical University of Warsaw, Warsaw, Poland; 3 Department of Microbiology, Medical University of Warsaw, Warsaw, Poland; 4 Department of Neurology, Medical University of Warsaw, Warsaw, Poland; 5 University Clinical Center of Medical University of Warsaw, Medical University of Warsaw, Warsaw, Poland; 6 Department of Adult Infectious Diseases, Medical University of Warsaw, Warsaw, Poland; Loyola University Health System, UNITED STATES

## Abstract

Multiple sclerosis (MS) is a chronic, immune-mediated demyelinating disease of the central nervous system of unclear etiology, but there is some evidence that viral infections could be responsible for triggering autoimmune mechanisms against myelin. We searched for viral RNA and DNA in cerebrospinal fluid (CSF) of 34 MS patients and 13 controls using RT-PCR/PCR against common neurotropic viruses. In addition, shotgun DNA- and RNA-based metagenomics were done in 13 MS patients and 4 controls. Specific quantitative real-time RT-PCR/PCR testing revealed the presence of viral nucleic acid in seven (20.59%) MS patients and in one (7.69%) control patient. In MS patients the most frequently detected was human herpesvirus type 6 (HHV-6; 3 cases; 8.82%); followed by Epstein-Barr virus (EBV; 2 cases; 5.88%), varicella zoster virus (VZV; 1 case; 2.94%) and Enterovirus (EV; 1 case; 2.94%). The single identified virus among controls was EBV (7.69%). DNA and RNA metagenomic assays did not identify any known eukaryotic viruses even though three of the analyzed samples were low-level positive by specific quantitative real-time PCR. In conclusion, we detected the presence of *Herpesviridae* and occasionally *Enteroviridae* in CSF from patients with MS but their prevalence was not significantly higher than among controls. Metagenomic analysis seems to be less sensitive than real-time RT-PCR/PCR and it did not detect any potential viral pathogens.

## Introduction

Multiple sclerosis (MS) is a chronic, immune-mediated demyelinating disease of the central nervous system (CNS); [1]. The Global Burden of Diseases, Injuries, and Risk Factors Study estimated that the number of patients worldwide is close to 2.2 million which poses a major health and economic burden on the society [2]. In the US it is estimated that the advanced stages of the disease result in healthcare costs ranging from $8,528 to $54,244 per patient per year [3]. Unfortunately, despite intensive research the pathogenesis of the disease remains unknown [4].

A number of patient factors have been suspected to play some role in MS development, including genetic susceptibility [5], high estrogen levels [6], smoking [7] and vitamin D deficiency [8], but there is also evidence for environmental influence since there is an increase in disease risk for all individuals moving from low to high MS prevalence areas [9].

A number of epidemiological studies have linked various viruses including Epstein-Barr virus (EBV), human herpesvirus type 6 (HHV-6), human cytomegalovirus (CMV), herpes simplex viruses (HSV), human endogenous retrovirus (HERV), Measles virus (MeV) and even transfusion transmitted virus (TTV) with MS but the strongest overall seems to be the association with EBV and HHV-6 [7, 9–11]. However, previous studies used routine diagnostic methods thus limiting the detection to the most common pathogens [12]. Next generation sequencing (NGS) metagenomics offers an alternate approach to the identification of pathogens as it allows for characterization of whole microbial communities [13].

Only a few metagenomic studies were conducted so far on viral populations in MS patients which resulted in finding of GB virus-C (GBV-C) in the brain of one MS patient [14] and a very limited number of NGS reads mapping to EBV, CMV, and parvovirus in cerebrospinal fluid (CSF) [15]. In our previous small study shotgun metagenomic analysis of CSF detected VZV-DNA in a patient with Central Nervous System Idiopathic Inflammatory Demyelinating Disorder (IIDD); [16]. This finding encouraged us to conduct a more comprehensive search for viral agents in CSF of MS patients using both RT-PCR/PCR testing for the most common neurotropic viruses and shotgun DNA/RNA-based metagenomics.

## Methods and methods

### Patients

Forty-seven patients (30 women, 17 men) aged from 17 to 71 years (median 30 years), who were admitted to the Department of Neurology, Medical University of Warsaw in the years 2012–2016 with suspicion of MS were subjects of the study. MS was eventually confirmed in 34 patients and excluded in the remaining 13 patients, who were considered controls. Patient and control demographical, clinical and laboratory data are shown in Table 1.

**Table 1:** Clinical data and RT-PCR/PCR results of multiple sclerosis (MS) patients and controls.

	MS	Controls
	n = 34	n = 13
**Age** (years; mean ± SD)	38.38 ± 13.85	31.23 ± 8.92
**Gender**:		
Male, n (%)	14 (41.17)	3 (23.07)
Female, n (%)	20 (58.82)	10 (76.9)
**At admission**:		
Visual symptoms, n (%)	5 (14.71)	3 (23.07)
Brainstem symptoms, n (%)	3 (8.82)	1 (7.69)
Sensory symptoms, n (%)	8 (23.53)	4 (30.76)
Gait and equilibrium disturbances, n (%)	3 (8.82)	2 (15.38)
Retrobulbar optic neuritis, n (%)	3 (8.82)	0 (0)
Brainstem syndrome, n (%)	3 (8.82)	4 (30.76)
Cerebellar syndrome, n (%)	2 (5.88)	1 (7.69)
Paresis, n (%)	10 (29.41)	0 (0)
**MRI findings**:		
Demyelinating lesions in MRI brain, n (%)	25 (73.53)	0 (0)
Demyelinating lesions in cervical spine MRI, n (%)	25 (73.53)	1 (7.69)
Demyelinating lesions in thoracic spine MRI, n (%)	11 (32.35)	0 (0)
**CSF analysis**:		
Cytosis (in 1μl), mean ± SD	5.73 ± 5.32	2.70 ± 1.97
% of lymphocytes, mean ± SD	59.43 ± 24.24	43.38 ± 23.66
% of monocytes, mean ± SD	40.48 ± 24.13	54.87 ± 24.30
Proteins (mg/dl), mean ± SD	37.78 ± 16.41	33.18 ± 12.26
Unique oligoclonal bands, n (%)	20 (58.82)	0 (0)
**Detected viruses, n (copies/ml)**:		
Human herpesvirus type 6	3 (900; 1100; 1150)	0
Epstein-Barr virus	2 (1650; 1750)	1 (1650)
*Enteroviruses*	1 (1500)	0
Varicella zoster virus	1 (550)	0

Four control patients were diagnosed with peripheral neuropathy, three had peripheral vertigo and two suffered from retinopathy, while the remaining four patients remained undiagnosed. MS was diagnosed according to the revised (2010) McDonald criteria [17], which were in use at the time of the study, but all our patients met the revised McDonald criteria introduced in 2017 [18]. All MS patients had Relapsing-Remitting MS (RRMS) and CSF was collected within 1–4 weeks from the onset of symptoms. After the initial hospitalization for 7–10 days during which the SM diagnosis was made, patients were followed up at the outpatient clinic for at least 2 years.

All patients gave a written informed consent and all research was performed in accordance with the relevant guidelines and regulations. The study was approved by the Internal Review Board of the Medical University of Warsaw (approval number: KB/8/2015).

### Nucleic acids extraction

CSF samples were centrifuged at 1,200 rpm for 20 min at 4°C, aliquoted and kept frozen at -80°C until analysis. For RT-PCR/PCR assays total RNA and DNA were extracted from 200μl of CSF using TRIzol LS (Thermo Fisher Scientific, USA) and NucleoSpin Plasma XS (Macherey-Nagel, Germany), respectively, whereas for metagenomic analysis nucleic acids were extracted from 500μl of CSF. Extracted RNA/DNA were suspended in 20μl of water.

### Virus-specific RT-PCR/PCR

CSF samples were analyzed using in-house quantitative real-time RT-PCR/PCR described previously [19–22]. These assays detected the following viruses: herpes simplex virus types 1 and 2 (HSV-1 and HSV-2, respectively), varicella zoster virus (VZV), EBV, CMV, HHV-6, human herpes virus type 7 (HHV-7), human adenoviruses (HAdVs) and enteroviruses (EV; Coxsackie A9, A16, B2, B3, B4, B5; ECHO 5, 6, 9, 11, 18, 30 and enterovirus 71). Limits of detection (LOD) for quantitative PCRs were as follows: for HSV-1–253 viral copies/ml, HSV-2–369 viral copies/ml, VZV—150 viral copies/ml, CMV– 403 viral copies/ml, EBV—226 viral copies/ml, HHV-6–111 viral copies/ml, HHV-7–153 viral copies/ml, HAdV—102 viral copies/ml and EV—240 viral copies/ml.

### DNA/RNA preamplification and NGS library preparation

Due to the expected low amounts of DNA/RNA in CSF a preamplification step was introduced to enable the construction of NGS libraries for sequencing [23]. RNA was reversely transcribed by single-primer isothermal amplification (Ribo-SPIA), using Ovation RNA-Seq V2 system (NuGEN, San Carlos, USA) following manufacturer’s recommendation. DNA was preamplified using SeqPlex Enhanced DNA Amplification protocol (Sigma-Aldrich, USA). Preamplified cDNA and DNA were purified using Agencourt AMPure XP beads (Beckman Coulter, USA) at a ratio of 0.8 (cDNA/DNA mixture volume to beads).

Libraries for NGS were prepared from one ng of cDNA/DNA using Nextera XT Kit (Illumina, USA) following manufacturer’s protocol. The quality and average length of NGS library was assessed using Bioanalyzer and DNA HS kit (Agilent Technologies, USA). Samples were double indexed, pooled equimolarly and sequenced on Illumina MiSeq (150nt, paired-end reads) or Illumina HiSeq (101nt, paired-end reads).

### NGS data analysis

Raw reads were trimmed in a process including adaptor and artifact removal and discarding reads with bases below quality score of Q20 (phred quality score) using Trimmomatic [24]. Reads shorter than 50 bp were excluded and the remaining reads were mapped to human reference sequence (hg19) with Stampy software [25]. Next, all unmapped sequences were compared to NCBI genomic viral reference database (viral RefSeq release 96) using Bowtie2 [26]. Reads were sorted, indexed with SAMtools [27], counted and statistically analyzed with standard R packages. Non-human sequences were uploaded into Sequence Read Archive (SRA); (BioProject ID PRJNA656949).

The criteria for positive virus detection were as follows: i) at least three reads specific for a particular viral species, ii) reads had to be distributed over the whole genome, iii) no presence of any of these viral reads in the control samples. Similar criteria for metagenomic virus detection were previously applied by others [28].

## Results

### Quantitative real-time RT-PCR/PCR

Specific quantitative real-time PCR testing revealed the presence of viral nucleic acid in seven (20.59%) out of 34 MS patients and in one (7.69%) out of 13 control patients (not significant by Fisher's exact test). The most frequently detected virus in MS patients was HHV-6 (3 cases; 8.82%); followed by EBV (2 cases; 5.88%), VZV (1 case; 2.94%) and EV (1 case; 2.94%). The single identified virus among controls was EBV (7.69%). CSF viral loads ranged from 550 to 1750 copies/ml (Table 1).

### DNA/RNA metagenomics

Metagenomic analysis was conducted on CSF samples from 13 MS patients and 4 controls. In the remaining cases either not enough CSF sample was left for analysis, or the required amount of cDNA/DNA to generate libraries for sequencing could not be obtained. Three of these patients were positive by real-time PCR. All 17 CSF samples underwent RNA-based metagenomic, while DNA workflow was limited to 16 since in one sample the amount of DNA generated was insufficient for NGS library construction.

After quality control DNA sequencing provided 211,440,331 reads (average 13,215,021 reads per sample) while the RNA approach provided 451,782,975 reads (mean 26,575,469 reads per sample). Detailed metagenomic data are shown in Table 2.

**Table 2:** Results of next-generation sequencing (NGS) metagenomic analysis of cerebrospinal fluid samples from 13 patients with multiple sclerosis (MS) and 4 controls. Reads were compared to the NCBI genomic viral RefSeq database (release 96).

	Pt.ID.	DNA metagenomics			RNA metagenomics			PCR results (copies/ml)
		Reads after trimming	Human reads	Viral reads	Reads after trimming	Human reads	Viral reads	
			%	%		%	%	
Patients with multiple sclerosis	**Pt.1**	11,259,882	11,102,501	543	28,346,849	27,576,271	2,609	_
			98.6023%	0.0048%		97.2816%	0.0092%	
	**Pt.2**	11,652,912	11,585,099	151	35,374,714	34,774,984	2,650	_
			99.4181%	13%		98.3046%	0.0075%	
	**Pt.3**	9,452,381	9,425,323	42	28,264,498	27,371,137	69,061	EBV
			99.7137%	0.0004%		96.8393%	0.2443%	1650
	**Pt.4**	10,199,344	10,110,897	112	32,157,586	30,886,056	3,649	_
			99.1328%	0.0011%		96.0459%	0.0113%	
	**Pt.5**	--	--	--	30,341,514	27,908,574	8,134	_
						91.9815%	0.0268%	
	**Pt.6**	11,450,725	11,290,934	152	24,362,412	21,334,870	14,492	HHV-6
			98.6045%	0.0013%		87.5726%	0.0595%	900
	**Pt.7**	12,497,288	12,296,580	206	34,597,626	30,625,349	10,965	_
			98.3940%	0.0016%		88.5186%	0.0317%	
	**Pt.8**	9,683,936	9,661,702	27	27,863,972	25,171,399	17,415	_
			99.7704%	0.0003%		95.0061%	0.0625%	
	**Pt.9**	11,356,129	11,286,777	89	29,526,092	28,051,587	3,859	_
			99.3893%	0.0008%		92.045%	0.0131%	
	**Pt.10**	14,047,954	13,841,856	142	32,263,205	30,998,982	2,647	_
			98.5329%	0.0010%		96.0815%	0.0082%	
	**Pt.11**	17,996,325	17,970,847	3,847	16,143,084	15,556,034	5,190	_
			99.8584%	0.0214%		96.3635%	0.0321%	
	**Pt.12**	17,316,819	17,243,319	1,812	15,986,092	15,665,630	3,520	VZV
			99.5756%	0.0105%		97.9954%	0.0220%	550
	**Pt.13**	18,198,157	17,928,811	4,596	14,837,915	14,524,058	3,926	_
			98.5199%	0.0253%		97.8848%	0.0265%	
Controls	**C1**	11,477,569	11,455,335	10,174	37,763,528	35,475,114	15,440	_
			99.8063%	0.0886%		93.9401%	0.0409%	
	**C2**	16,844,204	16,574,858	2,074	15,670,873	15,357,016	5,691	_
			98.4010%	0.0123%		97.9900%	0.0363%	
	**C3**	17,059,233	15,133,657	6,352	15,423,599	14,487,706	5,923	_
			88.7124%	0.0372%		93.9321%	0.0384%	
	**C4**	10,947,473	10,715,422	346	32,859,416	27,789,872	54,254	_
			97.8803%	0.0032%		84.5720%	0.1651%	

Regardless of the applied metagenomic workflow, the vast majority of NGS reads mapped to human genome (mean: 96.21%). DNA sequencing protocol provided from 42 to 10,174 (0.0003–0.0886%, mean: 0.0132%) viral reads, whereas RNA metagenomics provided from 2,609 to 69,061 (0.0075–0.2443%; mean: 0.0491%) reads mapping to viral database. The vast majority of identified viruses were bacteriophages, whereas the remaining viral reads either did not fulfil the criteria for positivity or were contaminants and artifacts (not shown). Applying the initially established criteria, no eukaryotic viruses were detected in MS patients or controls.

## Discussion

Viral infections are likely to play an important role in the pathogenesis and exacerbation of MS as evidenced by epidemiological studies and a number of reports on the potential of viruses to trigger autoimmune responses to such mechanisms as myelin by molecular mimicry, epitope spreading and bystander effect [10, 12, 29, 30]. Virus caused demyelination is a known phenomenon and has been previously described for Progressive Multifocal Leukoencephalopathy in which JC virus infects and ultimately kills myelin-producing oligodendrocytes [31]. A similar mechanism could be operational for canine distemper virus (CDV) infection in the white matter of dogs [32]. However, as *Herpesviridae* cause chronic infection it cannot be excluded that their presence in CSF from MS patients is the result of their reactivation [33, 34].

In the current study we identified four different viral species in CSF from patients with MS and the most frequently detected was HHV-6 as it was found in 3 patients (8.82%). Several earlier studies reported on the increased prevalence of HHV-6-DNA and anti-HHV-6 IgG/IgM in CSF of MS patients [35–38]. It has been proposed that HHV-6 might trigger demyelination by molecular mimicry of the virus-encoded U24 protein to myelin basic protein (MBP), which is a putative MS-associated autoantigen [39]. However, it should be noted that correlation between HHV-6 infection and MS was not confirmed in some other studies but the numbers of patients and controls were small [40–42].

A number of studies showed positive correlation between mononucleosis and MS [43–45] and thus detection of EBV DNA in two of our patients was not unexpected. It was calculated that mononucleosis increases the risk of MS development 2.3 times and in case of HLA-DR2-positive patients the risk is even 7 times higher [44].

VZV, another Herpesviridae was detected in one MS patient. VZV DNA was reported to be commonly present in CSF during MS relapses [46] and in our previous study we detected it in a patient with clinically isolated syndrome (CIS) which is considered to represent the earliest stage of MS demyelination [16, 47].

Enteroviral RNA was detected in one out of 34 MS patients. While *Enteroviruses* are typically associated with encephalitis, some enteroviral infections result in acute disseminated encephalomyelitis (ADEM), and hallmark of this syndrome is the presence of demyelination lesions in the brain and spinal cord [48, 49]. The patient was a 20 years-old male hospitalized because of sensory symptoms, but he admitted to having a short episode of diplopia 4 months earlier. A month after the current hospitalization the patient was readmitted because of retrobulbar optic neuritis. His symptoms were not preceded by any infection, he did not have fever, encephalopathy or headache. His MRI showed a symmetric pattern of T2-weighted hyperintense lesions in brain and spine including periventricular location. His CSF showed the presence of unique oligoclonal bands while pleocytosis and protein concentration were normal. The relapsing-remitting course, presence of unique oligoclonal bands in CSF and character of MRI changes strongly suggest that the patient had SM and not ADEM. Nevertheless, it cannot be excluded that the presence of enteroviral RNA in this case was coincidental and did not have any relationship to MS.

NGS-based metagenomic analysis offers a universal pathogen detection and has already been used to identify viruses in neuroinfections [50, 51]. However, despite our two-pronged RNA and DNA approach with a preamplication step no eukaryotic viruses were detected even though three of the analyzed samples were positive for *Herpesviruses* by specific real-time PCR. This discrepancy could be due to the fact that metagenomic workflows are less sensitive than specific real time RT-PCR/PCR assays and thus may fail in analysis of low viral-copy CSF samples [52]. Using serial dilutions of HIV and HSV positive sera in negative CSF, we have previously found that the limit of detection was 10^2^ and 10^3^ copies per reaction, respectively, while in the current study viral load in metagenomics-negative real-time PCR-positive samples ranged from 550 to 1650 copies/ml [53].

Although CSF is considered to be free of microbial DNA, we detected numerous reads mapping to various viral reference genomes but not fulfilling the initially established criteria of positivity. These mostly represented phage species and artifact sequences, which are particularly common for samples with a low DNA and RNA loads [54, 55]. Moreover, the reagents themselves could be the source of contaminating foreign sequences and may affect the interpretation of metagenomic results [53, 56].

## Conclusions

In conclusion, we detected the presence of *Herpesviridae* and occasionally *Enteroviridae* in CSF from MS patients but their prevalence was not significantly higher than among controls. Metagenomic analysis seems to be less sensitive than real-time RT-PCR/PCR and it did not detect any additional potential viral pathogens.
